# Burden and Risk Factors for Coronavirus Infections in Infants in Rural Nepal

**DOI:** 10.1093/cid/ciy317

**Published:** 2018-04-16

**Authors:** S M Iftekhar Uddin, Janet A Englund, Jane Y Kuypers, Helen Y Chu, Mark C Steinhoff, Subarna K Khatry, Steve C LeClerq, James M Tielsch, Luke C Mullany, Laxman Shrestha, Joanne Katz

**Affiliations:** 1 Department of International Health, Johns Hopkins Bloomberg School of Public Health, Baltimore, Maryland; 2 Seattle Children’s Hospital and Research Institute, Seattle; 3 Molecular Virology Laboratory, School of Medicine, Seattle; 4 Harborview Medical Center, University of Washington, Seattle; 5 Global Health Center, Cincinnati Children’s Hospital Medical Center, Ohio; 6 Nepal Nutrition Intervention Project–Sarlahi, Kathmandu; 7 Department of Global Health, Milken Institute School of Public Health, George Washington University, D.C; 8 Department of Pediatrics and Child Health, Institute of Medicine, Tribhuvan University, Kathmandu, Nepal

**Keywords:** acute respiratory infection, infants, coronavirus, respiratory virus, epidemiology

## Abstract

**Background:**

Knowledge of risk factors for symptomatic human coronavirus (HCoV) infections in children in community settings is limited. We estimated the disease burden and impact of birth-related, maternal, household, and seasonal factors on HCoV infections among children from birth to 6 months old in rural Nepal.

**Methods:**

Prospective, active, weekly surveillance for acute respiratory infections (ARIs) was conducted in infants over a period of 3 years during 2 consecutive, population-based randomized trials of maternal influenza immunization. Midnasal swabs were collected for acute respiratory symptoms and tested for HCoV and other viruses by reverse-transcription polymerase chain reaction. Association between HCoV incidence and potential risk factors was modeled using Poisson regression.

**Results:**

Overall, 282 of 3505 (8%) infants experienced an HCoV ARI within the first 6 months of life. HCoV incidence overall was 255.6 (95% confidence interval [CI], 227.3–286.5) per 1000 person-years, and was more than twice as high among nonneonates than among neonates (incidence rate ratio [IRR], 2.53; 95% CI, 1.52–4.21). HCoV ARI incidence was also positively associated with the number of children <5 years of age per room in a household (IRR, 1.13; 95% CI, 1.01–1.28). Of the 296 HCoV infections detected, 46% were coinfections with other respiratory viruses. While HCoVs were detected throughout the study period, seasonal variation was also observed, with incidence peaking in 2 winters (December–February) and 1 autumn (September–November).

**Conclusions:**

HCoV is associated with a substantial proportion of illnesses among young infants in rural Nepal. There is an increased risk of HCoV infection beyond the first month of life.

## Teaser

Human coronaviruses (HCoVs) are associated with respiratory illnesses in infants aged 0–6 months in a rural South Asian setting. HCoV incidence increases beyond the first month of life. Four tested species (229E, OC43, NL63, HKU1) were present.

Acute respiratory infections (ARIs) contribute significantly to pediatric mortality and morbidity worldwide [1], resulting in 3.1 million deaths annually [2]. Pneumonia, a severe manifestation of ARI, is the single largest cause of death in children worldwide, accounting for 16% of all deaths among children <5 years of age [3]. Viruses are the leading cause of ARIs, accounting for up to 80% of all cases [4, 5]. Increased use of polymerase chain reaction (PCR) over the past decade has led to a greater detection of previously underrecognized viruses such as human coronaviruses (HCoVs). HCoVs have been detected worldwide from persons of all ages [6]. HCoVs are commonly associated with upper respiratory tract infections, cause up to one-third of all common colds [7, 8], and can also cause severe lower respiratory tract infections, including pneumonia, in susceptible individuals [9–11]. There are 4 genera: alpha, beta, gamma, and delta coronaviruses. In the genus *Alphacoronavirus*, there are several species including HCoV-229E and HCoV-NL63. In the genus *Betacoronavirus*, species include HCoV-HKU1, HCoV-OC43, Middle East respiratory syndrome (MERS) coronavirus, and severe acute respiratory syndrome (SARS) coronavirus. HCoV-229E and HCoV-OC43 were first identified in the mid-1960s, with HCoV-NL63 and HCoV-HKU1 reported in 2004 and 2005, respectively. The 4 non-SARS/MERS species circulate widely in humans, whereas SARS-CoV and MERS-CoV are thought to spread in animal reservoirs, occasionally spilling over to human populations [6]. The SARS outbreak of 2002–2003 was caused by SARS-CoV, a novel coronavirus. This worldwide outbreak brought attention to this family of viruses; the discovery of HCoV-NL63 and HCoV-HKU1 followed shortly thereafter. The subsequent outbreak of MERS-CoV beginning in 2012 led to a renewed interest in HCoVs and their potential severity. Despite the growing interest and molecular characterization of these viruses, important gaps in knowledge about HCoVs remain. Limited data exist about the epidemiology of HCoVs in young infants and in persons of all ages in community settings, as well as the relative clinical importance of the 4 human HCoV species.

Here we examined disease burden and risk factors for HCoV-associated ARIs among children from birth to 6 months of age using a prospective, active home-based surveillance assessing the impact of maternal influenza vaccine on respiratory illnesses in rural Nepal [12].

## METHODS

We conducted a secondary analysis of data from 2 community-based, placebo-controlled, randomized trials of maternal influenza immunization, conducted in rural Sarlahi district, Nepal, from April 2011 to May 2014 [12]. Overall, 3693 pregnant women were enrolled in their second or third trimester and followed with weekly home-based visits from the time of vaccination to 6 months postpartum. Maternal and infant morbidity was assessed for the 7 days prior to each weekly household visit, and a midnasal sample was collected if an infant had any of the following criteria defining an ARI episode: fever, cough, difficult or rapid breathing, wheeze, or a draining ear [13]. Swabs were collected and placed in temperature-stable Primestore transport medium (Longhorn Diagnostics, Austin, Texas), and stored for up to a week at room temperature [12]. The tubes were then transported to the local laboratory, aliquoted into cryovials, and later shipped at room temperature to the University of Washington Molecular Diagnostics Laboratory in Seattle, where the samples were stored at –80°C. For HCoV detection, nucleic acids were extracted and tested using real-time reverse-transcription PCR (RT-PCR) with previously described methods [13, 14]. An HCoV ARI was defined as the presence of respiratory symptoms for a minimum duration of 1 day plus a midnasal swab positive for HCoV by RT-PCR. A single ARI episode was defined as an illness episode followed by 7 symptom-free days. The incidence of ARI episodes was calculated by dividing the number of cases by days at risk from birth to the end of study or loss to follow-up, and are reported per 1000 person-years (PY) of observation. Days with symptoms and the subsequent 7 symptom-free days (an ARI episode) were excluded from days at risk. A single swab from each episode was considered for the analysis. Therefore, the number of swabs was equal to the number of episodes.

Gestational age was estimated by calculating the difference between the date of birth and the last menstrual period (LMP). Date of LMP was obtained by visiting women of childbearing age every 5 weeks and asking for the date of LMP [12, 15]. Infant weights were measured as soon as possible after birth by trained study team members using a scale accurate to 10 g (Tanita BD-585). The weight was considered to be a birth weight if taken within 72 hours of birth. Low birthweight (LBW) was defined as <2500 g. Preterm birth was defined as birth before 37 completed weeks of gestation. Small for gestational age (SGA) was defined as birth weight <10th percentile for gestational age using the Intergrowth reference population [16]. A 4-category composite score was created using the preterm and SGA variables. For instance, children who were born at term and were appropriate for gestational age (AGA) were categorized as AGA term, and those who were born preterm and were SGA were categorized as SGA preterm. Breastfeeding initiation time was divided into within the first day (day 0) and beyond the first day of life. A neonate was defined as a child <28 days of age. Maternal smoking was defined as whether the mother smoked cigarettes or bidis (local hand-rolled cigarette) in the 30 days prior to enrolling in the trial. Households were defined as persons sharing a cookstove and household density was defined as the number of persons per household and number of children aged <5 years per room. A composite socioeconomic score estimating wealth of a household was created using multiple socioeconomic variables and those indicating household wealth measured by ownership of land, animals, and household items. A binary score was created for each item and summed to produce a score. Wealth quartiles were then created based on this score [17]. A composite indoor pollution score estimating pollution from indoor cookstoves was also created. Seasons were categorized as summer-monsoon (June–August), autumn (September–November), winter (December–February), and spring (March–May). Poisson regression was used to examine the association between birth-related, maternal, household, and external characteristics and HCoV respiratory illness in infants, using PY at risk of infection as the exposure. Mixed-effects modeling was used to account for correlated data of repeated episodes per child. Bivariate regression was performed first for each of the risk factors. Factors from the bivariate analysis were then included in the multivariate regression. Two different regressions were run, 1 where HCoV cases were compared to anyone without HCoV (non-HCoV ARI and those without ARI who may or may not have tested positive for HCoV if they had been tested) (model 1) and a second regression where HCoV cases were compared to all non-HCoV cases of ARI (ARI cases who tested negative for HCoV) (model 2). Logistic regression was used to examine any association between HCoV and other respiratory viruses as coinfections. All analyses were done with Stata 13.0 software (Stata Corp LP, College Station, Texas).

## RESULTS

**Figure 1.**
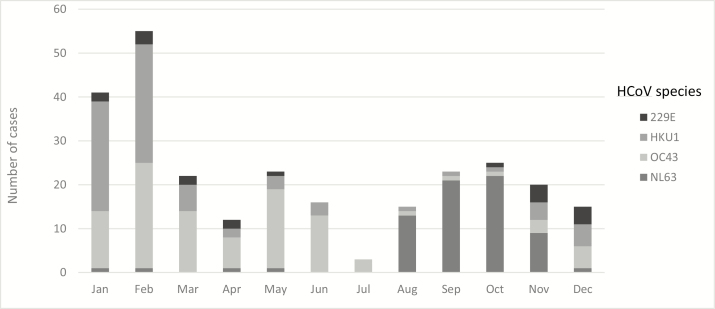
Number of human coronavirus (HCoV) cases over the study period, by HCoV species. Seasons are as follows: summer–monsoon, June–August; autumn, September–November; winter, December–February; spring, March–May.

A total of 3693 pregnant women were enrolled in the study from April 2011 to April 2013. Altogether, 3505 of the 3646 live-born infants were followed up for 1157.9 PY. A total of 4223 ARI episodes were detected in these infants, of which HCoV was detected in 296 swabs from 282 (8%) infants. These 296 HCoV-positive swabs included 103 swabs positive for HCoV-OC43, 78 positive for HCoV-HKU1, 70 for HCoV-NL63, and 19 for HCoV-229E. Species could not be determined for 26 HCoV-positive swabs. Fourteen repeat HCoV infections were also observed. Among these, 2 infants had 3 HCoV infections each in the course of the study. Median follow-up time overall was 175 days (interquartile range, 167–179 days) for an infant, and 53% of infants were followed through 180 days. Characteristics of the infants by HCoV and ARI status are shown in Tables 1 and 2. Overall HCoV incidence among infants was 255.6 (95% confidence interval [CI], 227.3–286.5) per 1000 PY. The incidence per 1000 PY of the HCoV species was as follows: HCoV-NL63: 59.6 (95% CI, 46.4–75.4), HCoV-OC43: 87.2 (95% CI, 71.1–106.0), HCoV-HKU1: 65.6 (95% CI, 51.7–82.2), and HCoV-229E: 16.4 (95% CI, 9.9–25.6). Seasonal variation of HCoV infections was observed, with the overall incidence peaking in 2 winters and 1 autumn. The highest incidence was observed in the autumn of 2011 at around 750 per 1000 PY (Supplementary Figure 1). Seasonality also varied among the HCoV species. HCoV-NL63 circulated every year of the study period and peaked around the autumn months. In contrast, HCoV-OC43 circulated in 2012 and 2013, and peaked around spring and late winter, respectively. Additionally, HCoV-HKU1 showed peak activity during winter of 2012, whereas HCoV-229E mainly occurred in early and late 2012 (Figure 1). Cough, fever, and wheeze were the most common clinical symptoms (Table 3). No differences in clinical symptoms were noted among species, although HCoV-NL63 had slightly more symptoms than the other 3 species. Bivariate regression analysis showed the incidence among infants outside the neonatal period was more than double that of neonates (incidence rate ratio [IRR], 2.38; 95% CI, 1.53–3.71; (Tables 4 and 5 and Supplementary Figure 2). Crowding in a household (number of children <5 years of age per room) was also associated with an increased risk of HCoV infection (IRR, 1.13; 95% CI, 1.01–1.28). In addition, poorer households were seen to be at increased risk compared to wealthier households. An increased risk was observed among infants who were SGA and preterm compared to those who were AGA and at term, but this was not statistically significant. Being born in the monsoon season or infected between June and August lowered the risk of HCoV compared to other times of the year. No association was seen between HCoV incidence and LBW, maternal age, literacy, smoking status, caste, and the indoor pollution score. In a multivariate model comparing HCoV-positive cases with HCoV-negative individuals (model 1), the risk factors that were significant included infant age, the number of children <5 years of age per room, and birth outside the summer-monsoon season (Table 3). In the model comparing HCoV positive cases with other ARI cases that were HCoV negative, the factors that were significant were infant age and monsoon season birth. As season of birth and season of infection were highly correlated, season of birth was selected for inclusion in the multivariate models. Coinfections with at least 1 other respiratory virus in the swab was seen in 137 of 296 HCoV infections (46%). Among these 137 HCoV coinfections, 125 (91.2%) were in nonneonates. The most frequent respiratory viruses found with HCoV coinfections were human rhinoviruses (HRVs), bocavirus, and respiratory syncytial virus (RSV) with 80, 12, and 8 cases, respectively. Supplementary Table 1 shows results of bivariate and multivariate Poisson regressions to examine the association of respiratory viruses with HCoV. With the exception of bocavirus, we see a negative association between HCoV and other respiratory viruses, with the rates of HCoV being lower when these other viruses were present. The negative association was significant for RSV, HRV, and influenza viruses in bivariate analysis and remained significant after adjusting for other viruses in the sample and the season of infection. Altogether, 31 of the 282 (11%) infants with HCoV ARIs received medical care. Of these, 2 infants sought care from a primary health center, MBBS (Bachelor of Medicine, Bachelor of Surgery) doctor, or a hospital. No deaths were reported among the HCoV cases. In comparison, 424 of 2030 (20.9%) infants with non-HCoV ARIs were brought to medical attention; 59 received care from a primary health center, MBBS doctor, or a hospital.

**Table 1.** Categorical Characteristics by Human Coronovirus and Acute Respiratory Infection Status of Infants Aged 0–6 Months

Characteristic	HCoV ARI Cases (n = 296), No. (%); PY^a^; Incidence/1000 PY (95% CI)	Non-HCoV ARI Cases (n = 3927), No. (%); PY^a^; Incidence/1000 PY (95% CI)	Infants Without ARIs (N = 1389), No. (%)
Infant age			
Neonates	21 (7.1%); 177.5; 118.3 (73.2–180.9)	530 (13.5%); 177.4; 2987.0 (2738.0–3252.4)	189 (13.6%)
Nonneonates	275 (92.9%); 980.4; 280.5 (248.3–315.7)	3397 (86.5%); 979.6; 3467.7 (3352.1–3586.3)	1200 (86.4%)
Sex			
Male	164 (55.4%); 605.9; 270.7 (230.8–315.4)	2237 (57.0%); 605.4; 3694.8 (3543.3–3851.2)	694 (50.0%)
Female	132 (44.6%); 552.8; 238.8 (199.8–283.2)	1690 (43.0%); 552.5; 3059.1 (2915.0–3208.5)	695 (50.0%)
Preterm			
Term	261 (88.2%); 1018.3; 256.3 (226.2–289.4)	3421 (87.1%); 1017.5; 3362.0 (3250.3–3476.6)	1218 (87.7%)
Preterm	35 (11.8%); 140.4; 249.2 (173.6–346.6)	506 (12.9%); 140.4; 3605.2 (3297.9–3933.5)	171 (12.3%)
Birth weight			
Normal	190 (64.2%); 733.4; 259.1 (223.5–298.7)	2555 (65.1%); 732.8; 3486.4 (3352.5–3624.3)	764 (55.0%)
LBW	55 (18.6%); 229.2; 239.9 (180.7–312.3)	804 (20.5%); 229.1; 3509.6 (3271.2–3760.8)	258 (18.6%)
Breastfeeding initiation time			
≤1 d	242 (81.8%); 936.5; 258.4 (226.9–293.1)	3146 (80.1%); 935.9; 3361.6 (3245.1–3481.1)	1045 (102.3%)
>1 d	52 (17.6%); 216.7; 240.0 (179.2–314.7)	754 (19.2%); 216.6; 3481.7 (3237.6–3739.4)	341 (33.4%)
Primiparity			
Nonprimiparous	182 (61.5%); 708.6; 256.8 (220.9–297.0)	2457 (62.6%); 708.1; 3469.7 (3333.8–3609.6)	745 (53.7%)
Primiparous	113 (38.2%); 448.4; 252.0 (207.7–303.0)	1457 (37.1%); 448.1; 3251.9 (3087.0–3423.2)	643 (46.3%)
Maternal smoking			
Nonsmoker	286 (96.6%); 1121.1; 255.1 (226.4–286.4)	3762 (95.8%); 1120.4; 3357.8 (3251.4–3466.9)	1356 (97.8%)
Smoker	9 (3.0%); 35.8; 251.0 (114.8–476.6)	152 (3.9%); 35.8; 4242.8 (3595.2–4973.5)	32 (2.3%)
Maternal literacy			
Literate	158 (53.4%); 671.2; 235.4 (200.1–275.1)	2093 (53.3%); 670.8; 3120.2 (2988.0–3256.8)	863 (62.2%)
Not literate	138 (46.6%); 487.5; 283.1 (237.8–334.4)	1834 (46.7%); 487.1; 3765.1 (3594.7–3941.4)	526 (37.9%)
Household ethnicity			
Pahadi	152 (51.4%); 644.4; 235.9 (199.9–276.5)	2112 (53.8%); 644.0; 3279.6 (3141.2–3422.5)	767 (55.2%)
Madeshi	133 (44.9%); 471.2; 282.3 (236.3–334.5)	1672 (42.6%); 470.8; 3551.2 (3383.0–3725.6)	570 (41.0%)
Wealth quartile			
<25th quartile	107 (36.1%); 320.3; 334.0 (273.8–403.7)	1256 (32.0%); 320.0; 3924.7 (3710.7–4148.0)	331 (23.8%)
50th quartile	86 (29.1%); 357.4; 240.6 (192.5–297.2)	1263 (32.2%); 357.1; 3536.4 (3344.0–3736.9)	419 (30.2%)
75th quartile	65 (22.0%); 311.6; 208.6 (161.0–265.9)	915 (23.3%); 311.4; 2938.4 (2751.1–3135.1)	402 (28.9%)
>75th quartile	27 (9.1%); 126.7; 213.0 (140.4–310.0)	350 (8.9%); 126.7; 2763.2 (2481.2–3068.4)	187 (13.5%)
Monsoon birth			
Other season birth	218 (73.6%); 800.6; 272.3 (237.3–310.9)	2605 (66.3%); 800.1; 3256.0 (3132.2–3383.5)	979 (70.5%)
Monsoon birth	78 (26.4%); 358.1; 217.8 (172.2–271.9)	1322 (33.7%); 357.8; 3694.4 (3497.9–3899.0)	410 (29.5%)
Infection season			
Summer-Monsoon (June–Aug)	34 (11.5%); 247.0; 137.6 (95.3–192.3)	783 (19.9%); 246.9; 3170.8 (2952.6–3400.9)	
Autumn (Sept–Nov)	87 (29.4%); 310.0; 280.7 (224.8–346.2)	1290 (32.8%); 309.7; 4164.9 (3940.7–4398.5)	
Winter (Dec–Feb)	117 (39.5%); 338.0; 346.1 (286.3–414.8)	1019 (25.9%); 337.7; 3017.4 (2835.0–3208.6)	
Spring (Mar–May)	58 (19.6%); 263.7; 220.0 (167.0–284.4)	835 (21.3%); 263.5; 3168.7 (2957.4–3391.1)	

Abbreviations: ARI, acute respiratory infection; CI, confidence interval; HCoV, human coronavirus; LBW, low birth weight; PY, person-years.

^**a**^PY at risk.

**Table 2.** Continuous Characteristics by Human Coronavirus and Acute Respiratory Infection Status of Infants Aged 0–6 Months

Characteristic	HCoV ARI Cases (n = 296)	Non-HCoV ARI Cases (n = 3927)	Infants Without ARIs (n = 1389)
Gestational age, wk, mean (SD)	39.7 (2.5)	39.3 (2.6)	39.4 (2.5)
Birthweight, g, mean (SD)	2813.2 (642.1)	2796.8 (542.0)	2782.6 (452.8)
Maternal age at enrollment, y, mean (SD)	23.3 (4.8)	23.2 (4.9)	22.9 (4.5)
Indoor air pollution score, mean (SD)	25.7 (10.4)	26.0 (10.3)	24.9 (9.9)
Children <5 y of age per room, mean (IQR)	0.8 (0.2–1)	0.8 (0–1)	0.6 (0–1)
Persons per household, mean (IQR)	9.3 (6–11)	9.3 (6–12)	8.9 (6–11)

Abbreviations: ARI, acute respiratory infection; HCoV, human coronavirus; IQR, interquartile range; SD, standard deviation.

**Table 3.** Clinical Presentation by Human Coronavirus Species and Acute Respiratory Infection Status Among Infants 0–6 Months of Age

Clinical Presentation	HCoV-OC43 Episodes (n = 103)	HCoV-HKU1 Episodes (n = 78)	HCoV-NL63 Episodes (n = 70)	HCoV-229E Episodes (n = 19)	HCoV Episodes (n = 296)	Non-CoV ARI Episodes (n = 3927)
Fever	57 (55.3)	35 (44.9)	40 (57.1)	8 (42.1)	155 (52.4)	2123 (54.1)
Fever only	14 (13.6)	11 (14.1)	6 (8.6)	3 (15.8)	37 (12.5)	573 (14.6)
Cough	70 (68.0)	47 (60.3)	51 (72.9)	13 (68.4)	198 (66.9)	2436 (62.0)
Cough only	13 (12.6)	13 (16.7)	8 (11.4)	5 (26.3)	42 (14.2)	472 (12.0)
Wheeze	42 (40.8)	38 (48.7)	31 (44.3)	6 (31.6)	128 (43.2)	1794 (45.7)
Wheeze only	2 (1.9)	5 (6.4)	2 (2.9)	1 (5.3)	11 (3.7)	102 (2.6)
Difficulty breathing	36 (35.0)	31 (39.7)	30 (42.9)	5 (26.3)	112 (37.8)	1582 (40.3)
Difficulty breathing only	3 (2.9)	5 (6.4)	1 (1.4)	1 (5.3)	11 (3.7)	123 (3.1)
Drippy ear	4 (3.9)	1 (1.3)	3 (4.3)	1 (5.3)	10 (3.4)	205 (5.2)
Drippy ear only	0 (0.0)	0 (0.0)	0 (0.0)	1 (5.3)	1 (0.3)	73 (1.9)
Difficulty feeding	7 (6.8)	6 (7.7)	13 (18.6)	1 (5.3)	28 (9.5)	325 (8.3)
Difficulty feeding only	0 (0.0)	0 (0.0)	0 (0.0)	0 (0.0)	0 (0.0)	0 (0.0)
Symptom combinations						
Fever and cough only	19 (18.4)	6 (7.7)	13 (18.6)	3 (15.8)	45 (15.2)	459 (11.7)
Fever, cough, wheeze, and difficulty breathing only	5 (4.9)	7 (9.0)	4 (5.7)	1 (5.3)	19 (6.4)	307 (7.8)
Coinfection	38 (36.9)	29 (37.2)	40 (57.1)	10 (52.6)	137 (46.3)	0 (0.0)
Coinfection–neonates	5 (4.9)	2 (2.6)	9 (12.9)	0 (0.0)	21 (7.1)	530 (13.5)
Mean duration of episode, d	4.41	5.12	6.14	5.21	5.24	4.66

Data are presented as No. (%) unless otherwise indicated.

Abbreviations: ARI, acute respiratory infection; HCoV, human coronavirus.

**Table 4.** Bivariate and Multivariate Risk Factor Analysis for Human Coronavirus Incidence Among Infants Aged 0–6 Months

Risk Factor	Comparison With All ARI HCoV Negatives or Not Tested (Asymptomatic)	Multivariate Model 1: Comparison With All ARI HCoV Negatives or Not Tested (Asymptomatic)	Multivariate Model 2: Comparison With Non-HCoV ARIs
	Unadjusted IRR (95% CI)	Adjusted IRR (95% CI)	Adjusted IRR (95% CI)
Infants			
Neonates	Ref	Ref	Ref
Nonneonates	2.38^a^ (1.53–3.71)	2.53^a^ (1.52–4.21)	2.05^b^ (1.24–3.37)
Sex			
Female	Ref	Ref	Ref
Male	1.14 (.90–1.44)	1.31 (1.00–1.73)	1.05 (.81–1.37)
Gestational age (wk)	1.04 (.99–1.09)		
Birthweight (g)	1.00 (1.00–1.00)		
Preterm			
Term	Ref		
Preterm	0.97 (.68–1.38)		
Birth weight and term			
Normal	Ref		
LBW	0.93 (.69–1.25)		
AGA	Ref		
SGA	1.00 (.76–1.32)		
AGA and term	Ref	Ref	Ref
AGA and preterm	1.05 (.70–1.57)	0.92 (.61–1.39)	0.93 (.63–1.38)
SGA and term	0.99 (.74–1.32)	0.98 (.72–1.34)	0.99 (.74–1.33)
SGA and preterm	1.6 (.50–5.09)	1.56 (.48–5.09)	1.36 (.47–3.92)
Breastfeeding initiation time			
≤1 d	Ref	Ref	Ref
>1 d	0.93 (.68–1.27)	0.75 (.50–1.12)	0.78 (.53–1.15)
Maternal age at enrollment (y)	1.00 (.98–1.03)	1.01 (.97–1.05)	1.01 (.98–1.05)
Maternal smoking			
Nonsmoker	Ref	Ref	Ref
Smoker	0.99 (.52–1.89)	1.05 (.50–2.19)	0.87 (.44–1.70)
Primiparity			
Nonprimiparous	Ref	Ref	Ref
Primiparous	0.98 (.77–1.25)	1.16 (.79–1.70)	1.09 (.77–1.54)
Maternal literacy			
Not literate	Ref	Ref	Ref
Literate	0.83 (.66–1.05)	1.04 (.75–1.43)	1.17 (.86–1.59)
Household ethnicity			
Pahadi	Ref	Ref	Ref
Madeshi	1.20 (.94–1.52)	1.17 (.84–1.62)	1.20 (.87–1.64)
Persons per household	1.01 (.99–1.04)		
Children aged <5 y per room	1.18^b^ (1.07–1.30)	1.13^c^ (1.01–1.28)	1.05 (.93–1.18)
Wealth quartile			
<25th quartile	Ref	Ref	Ref
50th quartile	0.69^b^ (.51–.91)	0.72 (.51–1.00)	0.78 (.56–1.07)
75th quartile	0.60^b^ (.44–.83)	0.68 (.46–1.01)	0.81 (.56–1.17)
>75th quartile	0.62^c^ (.40–.96)	0.63 (.35–1.11)	0.79 (.46–1.34)
Indoor air pollution score	1.00 (.99–1.02)	1.00 (.98–1.01)	0.99 (.98–1.01)
Monsoon birth			
Other season birth	Ref	Ref	Ref
Monsoon birth	0.8 (.61–1.04)	0.66^c^ (.48–.91)	0.61^b^ (.45–.83)
Infection season			
Summer–monsoon infection (June–Aug)	Ref		
Autumn infection (Sept–Nov)	1.52^c^ (1.03–2.24)		
Winter infection (Dec–Feb)	2.47^a^ (1.71–3.58)		
Spring infection (Mar–May)	1.56^c^ (1.04–2.35)		

Covariates in models 1 and 2: infant age (neonate or not), sex, SGA, preterm, interaction between SGA and preterm, breastfeeding initiation time, maternal age, smoking, parity, literacy, ethnicity (Pahadi/Madeshi), number of children <5 years of age per room, wealth quartile, indoor air pollution score, season of birth.

Abbreviations: AGA, appropriate for gestational age; ARI, acute respiratory infection; CI, confidence interval; HCoV, human coronavirus; IRR, incidence rate ratio; LBW, low birthweight; SGA, small for gestational age.

^a^ *P* < .001.

^b^ *P* < .01.

^c^ *P* < .05.

**Table 5.** Human Coronavirus Incidence by Age of Infants

Infant Age, mo	HCoV Incidence, per 1000 PY
<1	137
1	264
2	290
3	293
4	279
5	264

Abbreviations: HCoV, human coronavirus; PY, person-years.

## DISCUSSION

Approximately 8% of infants experienced HCoV-associated ARIs within the first 6 months of life in a prospective home-based surveillance study in rural, southern Nepal. HCoV incidence was significantly higher among older infants than neonates, and was positively associated with the number of children aged <5 years per room and births during non–monsoon seasons. Overall HCoV incidence peaked during 2 winters and 1 autumn in the course of the study. All 4 species were present with overlap in timing of circulation. OC43 had the highest incidence, followed by HKU1, NL63, and 229E. The lower incidence among neonates could be explained by either social factors or the presence of high levels of maternal antibodies at birth against all types of HCoVs that decline within a few months [17]. In our study, the increased risk was seen from 2 months of age and did not increase over time thereafter, suggesting that if maternal antibodies played a role, they waned relatively quickly. An increase in HCoV infections with age was also seen in previous studies [18, 19]. Gagneur et al [20] found no association between HCoV infections with gestational age and birthweight among 64 neonates, of whom 7 had HCoV infections and 57 were uninfected. However, this was a hospital study examining nosocomial respiratory viral infections among neonates admitted to the neonatal intensive care unit.

In our study, HCoV incidence was positively associated with household crowding of young children. This has been observed by others [21, 22]. We did not observe any statistically significant associations between HCoV incidence and the other factors analyzed (infant sex, gestational age, birth weight, maternal age, literacy, smoking, parity, ethnicity, household wealth, presence of an indoor biomass stove). Our HCoV incidence was 255.6 per 1000 PY among study children. Previous studies of HCoV incidence in children have focused on different age groups, used a narrower definition of ARI, or had different follow-up schedules to our study. For example, HCoV incidence ranged from 77.1 per 1000 PY in Costa Rica to 17.6 per 1000 PY in Singapore among children aged 6 months to <10 years enrolled in an influenza vaccine efficacy trial [23]. The lower incidence reported in the trial could be explained by the inclusion of only febrile cases, conducting follow-up only every 1–2 weeks, and a wider age range. A study in Pakistan reported an HCoV incidence of 7.3 per 1000 PY in children aged 0–24 months with severe pneumonia under a fortnightly surveillance [24]. For comparison, when we repeated our analyses using only febrile cases, HCoV incidence was 82.3 per 1000 PY. HCoVs were found in 7% of all ARI episodes in our study, compared to 5%–18% in previous studies [24–27]. We believe that weekly surveillance for morbidity assessment in our study minimized recall bias, allowing for better capture of ARI episodes. HCoV infections were seen throughout the study period with >1 peak per year. Large year-to-year periodicity in HCoVs was also observed over 3 years of surveillance. HCoV incidence peaked in 2 winters and 1 autumn, and reached a nadir in 2 summer-monsoon seasons. Similar seasonal trends were reported in Western Europe and the United States [14, 19, 28]. Among the HCoV species, HCoV-NL63 was detected in every year of the study and peaked around the autumn months, comparable to the seasonality reported in Hong Kong [29]. Frequency of HCoV-NL63 was highest in 2011 and 2013, which suggests that this species peaks every other year, similar to the pattern reported in Western Europe [30]. HCoV-OC43 circulated around spring-winter of 1 study year, similar to the seasonality seen in Western Europe/United States [14, 19]; however, this species also showed peak activity in the spring. HCoV-HKU1 was predominant in winter whereas HCoV-229E showed no particular seasonality. We did not see notable differences in symptoms or duration of illness by species, similar to Ogimi et al in a hospital-based population [31]. Of the 296 HCoV infections, 137 (46%) were coinfections with at least 1 other respiratory virus detected in the swab. This compares to coinfection rates ranging from 11% to 73.5% in previous studies [14, 19, 23, 32, 33]. Rhinoviruses were the most frequently codetected viruses, followed by bocavirus and RSV. A study of children aged <14 years with lower ARI in China [32] found HRV to be the most commonly detected virus in HCoV coinfections. A study of children aged 0–19 years with ARI found RSV to be the most commonly detected virus in HCoV coinfections, but HRV was not tested in that study [14]. We also found an increased rate of coinfections among nonneonates compared to neonates (91.2% vs 8.8%). We found a negative association between HCoV and HRV, RSV, and influenza viruses that were statistically significant after adjusting for other viruses and season of infection. This suggests that an infection by any 1 of these viruses could make it less likely for an individual to be infected by an HCoV. A similar relationship between HRV and HCoV has been reported previously in healthy children aged 6–24 months [34] and in individuals with ARI, nearly half of whom were infants aged <1 year [35]. The negative association between the viruses could perhaps be explained by the induction of interferons and other cytokines following infection by 1 virus, which could then prevent infection by the subsequent virus [36, 37]. Despite active surveillance, our study has several limitations. Swabs from asymptomatic infants were not collected, which could underestimate HCoV carriage but overestimate incidence. Very few studies have conducted active surveillance for asymptomatic infections in young infants, although these studies have documented 4% and 28% of nasal specimens positive for coronavirus in the absence of symptoms [27, 38]. Background rates of asymptomatic HCoV infection would also enable calculation of attributable fraction. This would provide further evidence of the association of HCoV infections and ARIs in infants; a higher attributable fraction would imply a stronger association. In addition, subjective maternal report of infant fever was used as a symptom for ARI instead of measured temperature. However, a previous validation study in this area showed good sensitivity and specificity of maternal report of fever [39]. The study was conducted in a poor, rural district with specific demographics, thus potentially limiting generalizability. However, this region is similar to many rural areas of north India, Bangladesh, and Pakistan and is representative of a large population residing within South Asia. Active home-based surveillance of infants in a rural South Asian setting showed that HCoV was associated with a substantial proportion of illnesses among infants. Knowledge of HCoV infections is limited in community settings and there is also a lack of data on infection risk factors. This study fills a gap in our knowledge of the epidemiology of coronaviruses, specifically in a rural, population-based setting in South Asia.

## Supplementary Data

Supplementary materials are available at *Clinical Infectious Diseases* online. Consisting of data provided by the authors to benefit the reader, the posted materials are not copyedited and are the sole responsibility of the authors, so questions or comments should be addressed to the corresponding author.

Supplementary Materials

## Notes

***Author contributions.*** J. K., M. C. S., J. M. T., J. A. E., and J. Y. K. contributed to the design of the study. J. Y. K.’s laboratory conducted the polymerase chain reaction analysis of specimens and subtyping of human coronaviruses. M. C. S. conceived the study and secured funding for the project. S. K. K., S. C. L., L. S., J. M. T., L. C. M., and J. K. supervised the conduct of the study in the field. S. M. I. U. assisted J. Y. K. in the subtyping analysis, drafted the manuscript, and designed/implemented the data analysis. H. Y. C. assisted in study implementation and cord blood collection. All authors read and approved the final manuscript.

***Disclaimer.*** The funding agency had no role in the design, conduct, analysis, and interpretation of the findings, or in the decision to publish.

***Financial support.*** This work was supported by a grant from the Bill & Melinda Gates Foundation (grant number 50274).

***Potential conflicts of interest.*** H. Y. C. has received research support from GlaxoSmithKline (GSK), Sanofi Pasteur, and Novavax. J. A. E. has been a consultant for Pfizer and a member of a data and safety monitoring board for GSK influenza antiviral studies, and her institution has received research support for clinical studies from GSK, Gilead, Chimerix, and Roche. All other authors report no potential conflicts of interest. All authors have submitted the ICMJE Form for Disclosure of Potential Conflicts of Interest. Conflicts that the editors consider relevant to the content of the manuscript have been disclosed.
