# Supplementary material for: Burden and Risk Factors for Coronavirus Infections in Infants in Rural Nepal
Source: Clin Infect Dis. 2018 Apr 16;67(10):1507–14. doi: 10.1093/cid/ciy317 (PMC6206108; doi:10.1093/cid/ciy317)
Supplement: Supplementary Materials [file ciy317_suppl_supplementary_materials.docx]

|  | **Supplemental Table 1:** HCoV co-infection frequencies and logistic regressions examining association between HCoV infection and other viral infections | | | | | |
| --- | --- | --- | --- | --- | --- | --- |
| **Respiratory viruses** | **Total number of infections^a^** | **HCoV – respiratory virus co-infection frequency ^a^** |  | **Crude OR (95% CI)** |  | **Adjusted OR (95% CI)** |
|  |  |  |  |  |  |  |
| **RSV** | 318 | 8 |  | 0.47** (0.27,0.84) |  | 0.38*** (0.21,0.68) |
| **MPV** | 177 | 5 |  | 0.84 (0.47,1.50) |  | 0.57 (0.32,1.03) |
| **PIV1** | 68 | 1 |  | 0.19 (0.03,1.33) |  | 0.12* (0.02,0.86) |
| **PIV2** | 38 | 0 |  | 0.98 (0.34,2.85) |  | 0.82 (0.26,2.57) |
| **PIV3** | 161 | 5 |  | 0.65 (0.32,1.28) |  | 0.57 (0.28,1.13) |
| **PIV4** | 67 | 2 |  | 0.38 (0.10,1.49) |  | 0.37 (0.10,1.43) |
| **ADV** | 78 | 3 |  | 0.67 (0.26,1.75) |  | 0.60 (0.23,1.54) |
| **RHV** | 1859 | 80 |  | 0.51*** (0.40,0.64) |  | 0.44*** (0.35,0.56) |
| **BOV** | 202 | 12 |  | 1.17 (0.74,1.85) |  | 1.16 (0.74,1.82) |
| **FLU** | 65 | 2 |  | 0.23* (0.08,0.72) |  | 0.18** (0.06,0.56) |
| **Seasons** |  |  |  |  |  |  |
| Summer-Monsoon |  |  |  |  |  | Ref |
| Autumn |  |  |  |  |  | 1.62* (1.10,2.39) |
| Winter |  |  |  |  |  | 2.26*** (1.57,3.25) |
| Spring |  |  |  |  |  | 1.49 (1.00,2.23) |
|  |  |  |  |  |  |  |
| ** p<0.05, ** p<0.01, *** p<0.001* |  |  |  |  |  |  |
|  | *^a^ Frequency = number of samples that was found positive for HCoV and the respiratory virus only Total co-infections = 159* | |  |  |  |  |


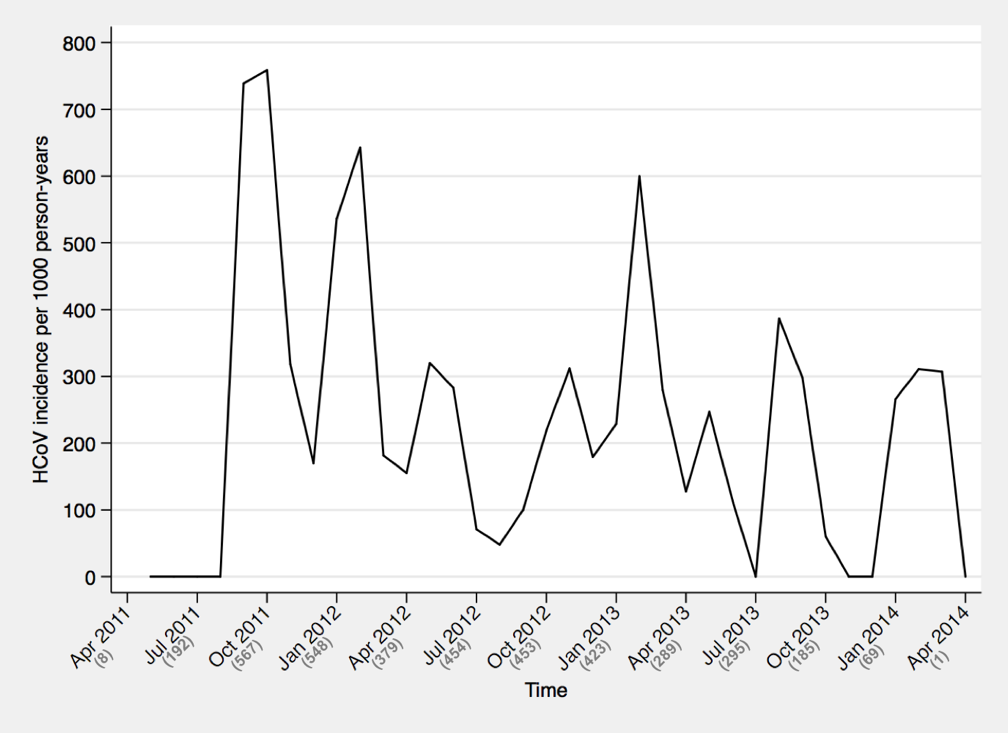


**Supplemental Figure 1:** Total HCoV incidence over study period. Summer-monsoon: June – August, autumn: September – November, winter: December – February and spring: March – May.


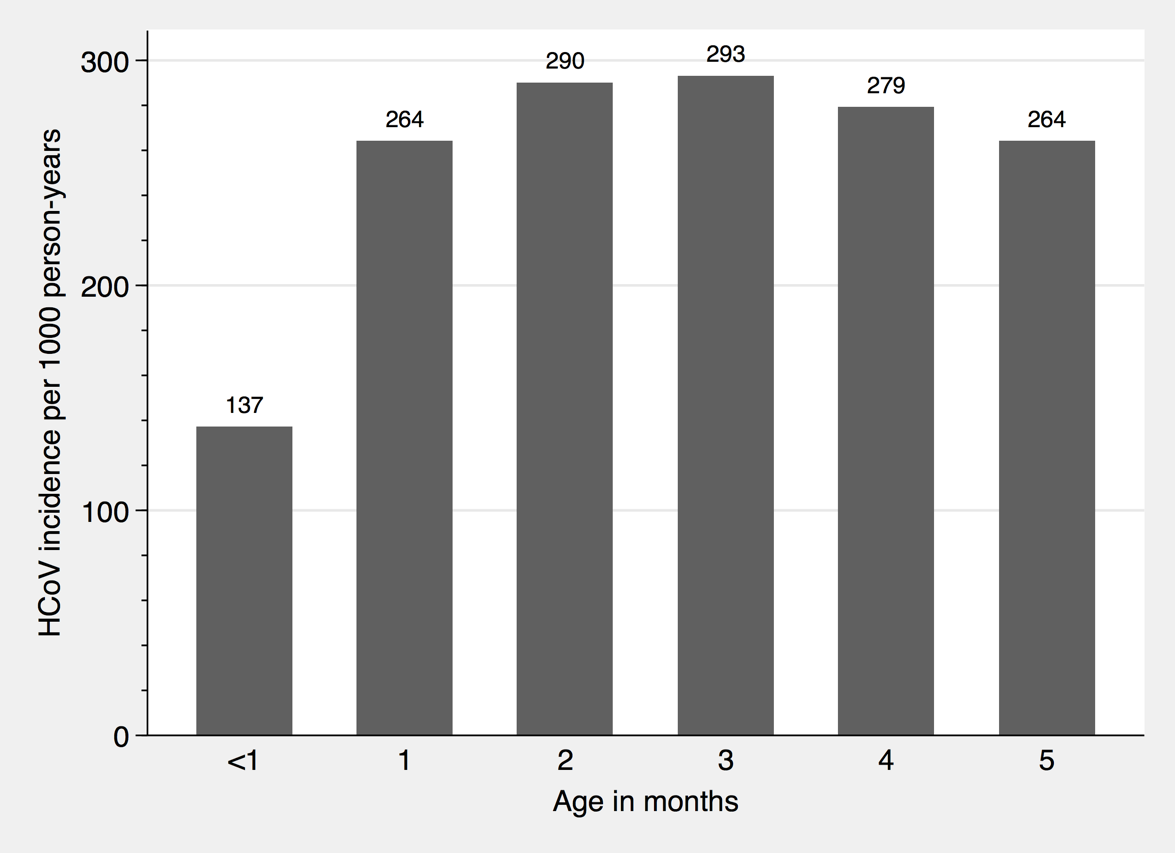

**Supplemental Figure 2:** HCoV incidence by age of infants.
